# Innovative biomaterials for the treatment of periodontal disease

**DOI:** 10.3389/fdmed.2023.1163562

**Published:** 2023-05-30

**Authors:** Yi Zhu, Chen Tao, Cynthia Goh, Annie Shrestha

**Affiliations:** ^1^ Faculty of Dentistry, University of Toronto, Toronto, ON, Canada; ^2^ Stomatological Hospital of Chongqing, Key Laboratory of Oral Diseases and Biomaterial Sciences, Chongqing Municipal Key Laboratory of Oral Biomedical Engineering of Higher Education, Chongqing, China; ^3^ Department of Chemistry, University of Toronto, Toronto, ON, Canada; ^4^ Department of Materials Science and Engineering, University of Toronto, Toronto, ON, Canada; ^5^ Department of Laboratory Medicine and Pathobiology, University of Toronto, Toronto, ON, Canada; ^6^ Department of Dentistry, Mt. Sinai Hospital, Toronto, ON, Canada

**Keywords:** periodontitis, injectable hydrogel, biomaterials, immune modulation, bone regeneration

## Abstract

Periodontitis is a multifactorial disease that involves the destruction of hard and soft tissues surrounding the tooth. Routine periodontal treatment includes mechanical debridement (surgical and non-surgical) and the systemic administration of antibiotics. In contrast, severe and chronic periodontitis involves aggressive tissue destruction and bone resorption, and the damage is usually irreversible. In these severe cases, bone grafts, the delivery of growth hormones, and guided tissue regeneration can all be used to stimulate periodontal regeneration. However, these approaches do not result in consistent and predictable treatment outcomes. As a result, advanced biomaterials have evolved as an adjunctive approach to improve clinical performance. These novel biomaterials are designed to either prolong the release of antibacterial agents or osteogenic molecules, or to act as immunomodulators to promote healing. The first half of this review briefly summarizes the key immune cells and their underlying cellular pathways implicated in periodontitis. Advanced biomaterials designed to promote periodontal regeneration will be highlighted in the second half. Finally, the limitations of the current experimental design and the challenges of translational science will be discussed.

## 1. Introduction

**Figure 1:**
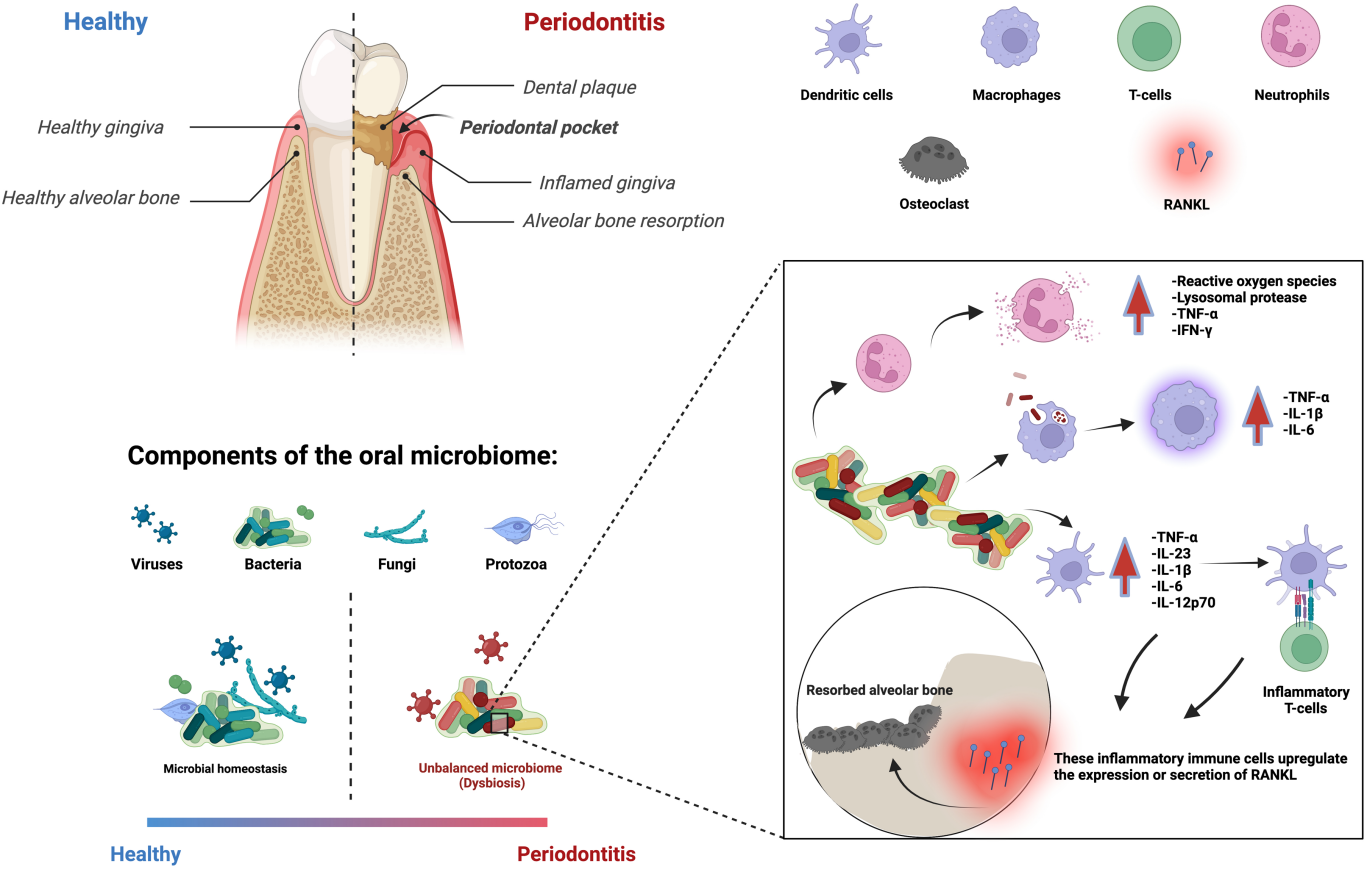
Periodontitis is an immune-inflammatory disease in response to bacterial biofilms that results in the loss of cementum, periodontal ligament, and bone resorption. Multiple immune cells are involved in this process.

Periodontitis is a multifactorial inflammatory disease that affects nearly 20%–50% of the global population (1). Its clinical signs include the inflammation and destruction of the periodontium, the supporting structure of the tooth composed of gingiva, periodontal ligament, cementum, and alveolar bone (2). Disruption in the equilibrium of bacterial biofilms can result in microbial dysbiosis, which can lead to oral diseases. Periodontal inflammation is associated with an increase in the prevalence of specific bacterial species, which triggers the recruitment of inflammatory immune cells to eradicate the pathogenic bacterial population (3–5). However, bacteria are difficult to eliminate when they are found in a dental biofilm, as they are protected by a 3D matrix of extracellular polymeric substances, which restricts the penetration of antibiotics and immune cells (6). As a result, the continuous interaction between microbial dysbiosis and the host immune response prolongs the inflammatory response. Moreover, immune cells and their associated pro-inflammatory cytokines not only target the bacteria but also activate the receptor activator of the NF-kB ligand (RANKL) pathway, which is responsible for osteoclastogenesis (the breakdown of bone tissue) (7) (Figure 1). Thus, both the host inflammatory response and bacterial collagenases contribute to tissue destruction and alveolar bone resorption (8, 9).

Furthermore, the effect of periodontitis extends beyond the oral cavity; numerous studies have associated periodontitis with systemic diseases, such as diabetes mellitus (10), cardiovascular disease (11), respiratory disease (12), and Alzheimer's disease (13). For instance, diabetes mellitus is considered a major risk factor for periodontitis, with an increased susceptibility of approximately threefold (14). The prevalence of periodontitis in diabetic patients was found to be close to 90% in a recent cross-sectional study (15). Although the exact mechanism between periodontitis and diabetes remains unclear, immune cells are known to be affected, resulting in worsened disease progression and delayed healing (16). Hyperglycemia, a hallmark of diabetes, is known to affect immune cells, resulting in chronic systemic inflammation (17, 18). Subsequently, this inflammatory state can negatively affect pancreatic cells and further reduce insulin production, which in turn negatively affects glycemic control, making it a vicious cycle (19). Moreover, activated inflammatory immune cells also contribute to inflammation-induced alveolar bone loss (20), leading to aggravated bone resorption in diabetic patients (10). Some studies have suggested that non-surgical interventions, such as scaling and root planing, can improve the clinical outcomes of diabetic periodontitis. A single-masked randomized clinical trial based on 90 patients has shown that scaling and root planing significantly improved periodontal parameters (pocket depth, gingival index, and plaque index) and metabolic parameters (serum glycated hemoglobin) compared to the group that did not receive scaling and root planing (21). These results are in good agreement with other clinical studies (22, 23). In addition, these data demonstrate that the counts of periodontal pathogenic bacteria correlate poorly with the clinical progression of diabetic patients, indicating that there are other factors at play other than bacterial-induced inflammation. Alternatively, insulin has been the gold standard to treat diabetes mellitus. In a recent study, it was suggested that insulin not only controlled blood glucose concentration, but it also suppressed inflammatory cytokines and ameliorated periodontitis without local periodontitis treatment in diabetic rats (24). These results further corroborate that the association between diabetes mellitus and periodontitis is indeed a two-way relationship.

Current periodontitis treatments range from non-surgical debridement (e.g., tooth scaling and root planing) to surgical debridement (flap procedure), combined with local or systemic antibiotic delivery to further inhibit bacterial activities (25). An alternate method to eliminate periodontal bacteria is known as photodynamic therapy (PDT). It is a non-invasive treatment that involves the use of a photosensitizer, which can be activated by a low-energy laser light to generate singlet oxygen and reactive oxygen species to eliminate microorganisms (26). Although *in vitro* antibacterial studies demonstrated PDT to be an effective approach against periodontal pathogens, such as *Porphyromonas gingivalis* (*P. gingivalis*) and *Aggregatibacter actinomycetemcomitans* (*A. actinomycetemcomitans*) (27, 28), systematic reviews of the utilization of PDT in clinical studies concluded that the efficacy of PDT as an adjunctive treatment is limited by the inadequate number of studies and the heterogeneity of study designs (29, 30). In most cases, scaling and root planing have been shown to be effective in attenuating periodontitis (31, 32). However, treatment outcomes vary based on factors such as the severity of periodontitis at baseline, the type of tooth affected, behaviors such as smoking and compliance, and other risk factors, such as diabetes mellitus, which leads to dysregulation of the immune system (33–36). However, while these treatments attenuate inflammation and preserve tissue loss, they do not actively promote the regeneration of periodontium.

In severe cases where there is substantial connective tissue and alveolar bone loss, surgical treatments such as bone grafts, delivery of growth hormones, and guided tissue/bone regeneration are recommended. These methods have shown some promise in promoting the regrowth of three-walled defects and narrow dehiscence defects (37). However, they are relatively ineffective at regenerating horizontal patterns of alveolar bone loss (38, 39). More recently, instead of delivering growth hormones, autologous platelet concentrates have been used. They are platelets isolated from patients’ own blood that are rich in growth factors and cytokines, such as platelet-derived growth factor (PDGF), transforming growth factor beta (TGFβ), vascular endothelial growth factor (VEGF), epithelial growth factor (EGF), insulin-like growth factor-1 (IGF-1), and basic fibroblast growth factor (bFGF), together with proteins such as fibrin, fibronectin, and vitronectin (40). These mediators regulate cell proliferation, migration, and differentiation, which are crucial in tissue regeneration and healing. Platelet-rich fibrin (PRF) is a well-studied second-generation derivative; its fibrin network functions as a three-dimensional scaffold that allows cell migration and the sustained release of growth factors. In one study, PRF was used as an adjunctive treatment combined with open flap debridement in a canine periodontitis model. The addition of PRF reduced the inflammation and improved soft tissue healing, but it did not affect alveolar bone gain (41). Another systemic review also reported that there is insufficient evidence that the addition of PRF significantly improved periodontal parameters such as probing pocket depth, clinical attachment level, and bone defect filling (40).

Given the intrinsic complexity of periodontitis and the inconsistent regenerative capability of current treatments, alternative or adjunctive treatments are constantly being explored. Regenerative strategies, such as the utilization of advanced biomaterials to promote periodontal regeneration, have emerged as viable alternatives. This review paper will first focus on the key immune cells involved in periodontitis, followed by the discussion of some of the latest biomaterial-based developments in treatment strategies to promote periodontal regeneration. The limitations of the current experimental designs, as well as the roadblocks to clinical translatability, are also discussed to provide some insight into the future development of innovative biomaterial-based periodontal therapies.

## 2. Immune cells and cellular pathways involved in periodontitis

Immune cells play an important role in alveolar bone homeostasis in chronic periodontitis (42). Neutrophils, mast cells, macrophages, dendritic cells (DCs), T-cells, and B-cells are among the innate and adaptive immune cells involved in this process. Under chronic microbial dysbiosis, the prolonged inflammatory states of these immune cells lead to periodontal disease progression and delayed healing (43).

### 2.1. Neutrophils

Polymorphonuclear leukocytes, also known as neutrophils, are one of the first responders to infections and play an important role in the innate immune system. They eliminate bacteria through a variety of key mechanisms, including phagocytosis (the ingestion and elimination of microbes), degranulation (the extracellular release of granules such as antimicrobial peptides), the production of reactive oxygen species (cytotoxic to both microbes and host cells), and neutrophil extracellular traps (trapping and eliminating pathogens while minimizing damage to host cells) (44).

Neutrophil homeostasis is critical for maintaining periodontal health; the absence or excess of neutrophils has been linked to aggravated periodontal inflammation (45, 46). Even in the absence of inflammation, neutrophils constantly infiltrate the periodontium in small quantities. These infiltrated neutrophils are also referred to as oral neutrophils, are frequently found in saliva and gingival crevicular fluid. Their number and activity are affected by periodontitis. For example, the presence of pathogenic dental plaque-derived chemoattractants and pro-inflammatory cytokines attracts more circulating neutrophils into the gingival tissues (47). A clinical study found an association between neutrophil cell counts and the severity of periodontitis (48). Patients with chronic periodontitis showed a 2.5-fold higher recruitment of neutrophils to their oral cavity when compared to healthy participants (49). Oral neutrophils are also functionally altered in periodontitis (50), characterized by dysfunctional chemotaxis with reduced speed, velocity, and chemotactic accuracy. In addition, neutrophils displayed increased levels of activation and degranulation markers such as CD10, CD63, CD64, and CD66a (47), as well as an altered transcriptome with a tendency to favor pro-survival genes, resulting in longer-lived neutrophils in periodontitis (51). The abundance and longevity of these neutrophils may impact the severity and length of inflammation-induced periodontal tissue destruction. These results indicate that neutrophils are chemotactically compromised, hyperactivated, and present in higher quantities in the presence of periodontitis.

Aside from their ability to fight microbial infections, neutrophils also exhibit plasticity. They can suppress the immune response via the suppression of T-cell activation (52). In one study, periodontal treatment reduced the number of suppressive neutrophils while increasing the number of normal neutrophils (53), implying that suppressive neutrophils may play a role in the etiology of periodontitis. However, due to the limited publications on the role of suppressive neutrophils in periodontitis, their exact contribution requires further investigation.

### 2.2. Mast cells

Mast cells are yet another important component of the innate immune response. They can be activated by several mechanisms, such as complement proteins, damage-associated molecular patterns (DAMPs), and pathogen-associated molecular patterns (PAMPs). Mast cells degranulate upon activation to induce inflammation in the local microenvironment. They secrete a variety of mediating cytokines, growth factors, and granules, such as tumor necrosis factor alpha (TNF-α), interleukin 1-beta (IL-1β), histamine, and monocyte chemoattractant protein (MCP-1), which all aid in the recruitment of other inflammatory cells and fibrocyte migration (54, 55).

The depletion of mast cells significantly reduced the production of inflammatory cytokines and periodontitis-induced alveolar bone loss in a rat experimental periodontitis model (56). A similar trend was reported in human gingival tissues (57), where the density of mast cells in periodontitis patients was 1.53-fold higher than in healthy patients. Another study compared healthy gingival tissues to groups with moderate and advanced periodontitis (58). The findings revealed an association between mast cell density, extent of degranulation, and severity of periodontitis. Furthermore, the expression of Toll-like receptor 4 (TLR4) on mast cells has been demonstrated to be positively correlated with the severity of chronic periodontitis (59). TLR4 is a sensing receptor for gram-negative bacteria that, when activated, induces a pro-inflammatory response (60).

### 2.3. Macrophages

Macrophages serve a vital role in both acute and chronic inflammation. Depending on their polarization, they can be either pro-inflammatory (M1) or anti-inflammatory (M2) (61). It is widely accepted that macrophages can adopt a continuum of phenotypes between these two distinct polarizations. Such intermediate macrophages could exhibit both M1 and M2 characteristics to different extents (62).

Resident oral mucosal macrophages adopt various phenotypes in the defense against periodontal pathogens. They also orchestrate the recruitment of monocytes (63, 64), which can differentiate into macrophages and dendritic cells. Environmental cues, such as the presence of periodontal pathogens, can influence macrophage polarization and PAMPs from bacteria can stimulate M1 macrophage differentiation, which establishes a pro-inflammatory environment and secretes pro-inflammatory cytokines such as IL-1β, IL-6, IL-23, and TNF-α (65, 66). In contrast, immunomodulatory cytokines such as IL-4, IL-13, and IL-10 are necessary to polarize macrophages toward the M2 phenotype (67, 68). Because M1 macrophages are associated with diseased tissues and M2 macrophages with healthy gingival tissues (69), an elevated M1/M2 ratio observed in periodontitis signifies an imbalance between inflammation and healing. Restoring this balance has been shown to be a potential treatment strategy for periodontitis. Recent research using bioactive molecules capable of inhibiting M1 differentiation effectively attenuated periodontal bone resorption in a murine periodontitis model (70). Similarly, another study found that injecting M2 macrophages into local periodontal tissue significantly alleviated the inflammation of periodontitis in mice (71).

Despite the findings that have associated M2 macrophages with the healing phase, many studies have reported contradictory results. In one study, it was observed that gingivitis tissues had the highest total number of macrophages as well as the highest M1 and M2 polarization than periodontitis and healthy samples (72); there was also no difference in the relative amounts of M1 and M2 macrophages between periodontitis and healthy tissues. Some of these discrepancies could be attributed to patient variability, differences in the analytical methods used to characterize and numerate the macrophage population, the timing of determining macrophage polarization, and the selection of surface markers, as some intermediate macrophages may exhibit characteristics of both M1 and M2.

### 2.4. Dendritic cells

Dendritic cells (DCs) are professional antigen-presenting cells that bridge the innate and adaptive immune responses in chronic periodontal disease (73, 74). They can elicit either immunogenic or tolerogenic immune responses depending on their activation state (75, 76). Immunogenic DCs activate inflammatory T-cells such as CD8+ T-cells (cytotoxic T-cells) and CD4+ T-cells (helper T-cells). Tolerogenic DCs, in contrast, have a limited capability to activate CD8+ T-cells and CD4+ T-cells. Rather, they stimulate regulatory T-cells (77), a subgroup of T-cells that suppress the immune system to maintain tolerance to self-antigens and prevent autoimmune disorders. In the case of periodontitis, inflammatory DCs can differentiate T-cells into a pro-inflammatory state. These activated CD4+ and CD8+ T-cells increase the expression and secretion of RANKL (78–80), which may facilitate osteoclastogenesis and contribute to inflammation-induced alveolar bone resorption.

Like macrophages, DCs can also recognize PAMPs presented by periodontal pathogens. Unstimulated DCs derived from the peripheral blood monocytes of patients with chronic periodontitis exhibited an immature phenotype, but their cytokine profiles indicated a proinflammatory response (81). This finding corroborates previous studies that have correlated chronic periodontitis with elevated systemic inflammation (82, 83). In another study, gingival samples from patients with chronic periodontitis showed higher levels of pro-inflammatory cytokines, such as IL-2, TNF-α, interferon gamma (IFN-γ), and IL-17A, when compared to healthy gingival tissues (84). Although the anti-inflammatory cytokine IL-10 was also elevated in the chronic periodontitis group, it did not alleviate periodontal inflammation. Moreover, clinical periodontal parameters (clinical attachment level >3) were correlated with a higher density of inflammatory DCs and negatively correlated with the density of immature DCs. Like other immune cells, inflammatory DCs can also be targeted to treat periodontitis. In one study, systemically administered antibiotics metronidazole and amoxicillin were given to patients with periodontitis; the treatment effectively reduced inflammatory DCs and improved the clinical measures of periodontitis (85). Aside from immunogenic DCs, tolerogenic DCs also serve a crucial role in immune regulation. They have been extensively investigated for their potential to maintain immunologic tolerance in clinical transplantation, autoimmunity, and inflammatory diseases (86–88). However, they have been underexplored as a possible therapeutic target for treating periodontitis.

### 2.5. T-cells

Oral mucosa constitutes resident T-cells, primarily memory CD4+ and CD8+ T-cells (89). Naïve T-cells from the lymph nodes can also be activated by antigen-presenting cells, such as DCs. When DCs encounter periodontal pathogens, they migrate to the lymph nodes, where naïve T-cells can be differentiated into various effector T-cells (74). The exact differentiation pathway depends on the type of DCs and the presence of immunomodulatory cytokines.

In periodontitis, two T-cell subsets have received much attention: regulatory T-cells and TH17 cells. Regulatory T-cells promote bone regeneration by downregulating the levels of TNF-α and IFN-γ. Moreover, they also secrete IL-4, a chemoattractant for osteoblasts (90). The adoptive transfer of CD8+ regulatory T-cells reduced alveolar bone destruction and osteoclast formation in experimental periodontitis (91, 92). In contrast, TH17 cells are associated with periodontitis (93). A murine experimental periodontitis model demonstrated that TH17 cells and the accumulation of neutrophils were necessary for inflammatory tissue destruction (94). In the same study, a patient population with a genetic defect in TH17 cell differentiation was associated with reduced periodontal inflammation and bone loss, despite an increased prevalence of recurrent oral fungal infections. These results emphasize the significance of TH17 cells in periodontitis.

The ratio of TH17 to regulatory T-cells has been linked to the health of periodontal tissues. Age was found to influence this ratio in blood samples from patients with chronic periodontitis (95). When compared to older patients, the TH17/regulatory T-cells ratio was much higher in younger individuals. Furthermore, DCs were also overactivated (upregulation of the CD40 marker) in younger patients. Cell culture experiments demonstrated that DCs with a high expression of CD40 elevated the Th17/regulatory T-cells ratio. In addition, hyperactivated DCs secreted cytokines that inhibited the induction of regulatory T-cells (96). Although these cells were not isolated from the gingival tissues, other studies have linked periodontitis-induced local inflammation to systemic inflammation (82, 97).

Similar to the M1/M2 ratio, the TH17/regulatory T-cells ratio can also be altered to attenuate periodontitis. An experimental periodontitis model established in rats revealed that the amount of CD8+ regulatory T-cells from the gingival tissues did not differ significantly from that of healthy animals (92). However, the population of TH17 cells was significantly elevated in the periodontitis group, while the amount of CD4+ regulatory T-cells decreased significantly when compared to healthy animals. Adoptive transfer of CD8+ regulatory T-cells reduced alveolar bone resorption and osteoclast formation. These findings suggest that periodontitis alters the proportion of regulatory T-cells and TH17 cells. Restoring this ratio to a healthy range by inducing new regulatory T-cells can be an effective approach for promoting periodontal regeneration. It is crucial to point out that in the presence of periodontitis, regulatory T-cells could become functionally unstable and lose their anti-osteoclastogenic properties. In a murine experimental periodontitis model, *ex vivo* suppression capacity on osteoclastic differentiation was dramatically reduced in regulatory T-cells obtained from periodontally diseased animals compared to healthy controls (98). Like macrophages, T-cells can undergo phenotypic transition. In periodontitis, regulatory T-cells have been shown to be converted to TH17 cells (99, 100). Thus, it may be necessary to induce new populations of regulatory T-cells directly from naïve T-cells or to introduce regulatory T-cells by adoptive transfer. Alternatively, the conversion of regulatory T-cells to TH17 could be modulated.

### 2.6. B-cells/plasma cells

B-cells are another set of APCs that are activated by helper T-cells after encountering a foreign antigen. Memory B-cells or plasma cells are two specialized forms. Plasma cells provide immediate protection via the secretion of specific antibodies, whereas memory B-cells generate a faster and enhanced response upon subsequent encounters with the same antigen (101).

Like other immune cells, B-cells have important implications in periodontitis as well (102). Most of the B-cells found in healthy gingiva and gingivitis tissues were memory B-cells. In contrast, the major B-cell population in periodontitis was plasma cells, which were more prominent at the base of the periodontal pocket and scattered throughout the gingiva. Plasma cells residing in periodontitis tissue secrete IgG antibodies that are specific to periodontal pathogens, such as *P. gingivalis* and *A. actinomycetemcomitan*s. In addition, these plasma cells also express RANKL, which is involved in osteoclastogenesis. Following scaling and root planing, the B-cell population in periodontitis tissues shifted from primarily plasma cells to memory B-cells. This result implies that the plaque biofilm was responsible for the differentiation of B-cells into plasma cells in periodontitis tissues. Circulating B-cells are also altered in periodontitis patients, as these cells have higher levels of RANKL expression (103).

Plasma cells are also heterogeneous. IgG plasma cells can differentiate into IL-37 producing plasma cells and IL-35 and IL-37 co-producing plasma cells (104). IL-35 and IL-37 are both anti-inflammatory cytokines that have been demonstrated to inhibit osteoclast formation *in vitro* in a dose-dependent manner. Thus, it is reasonable to hypothesize that these two subsets of plasma cells could attenuate periodontitis-induced alveolar bone resorption via the inhibition of osteoclast formation. These findings suggest that B-cells are essential to maintain alveolar bone homeostasis.

**Figure 2:**
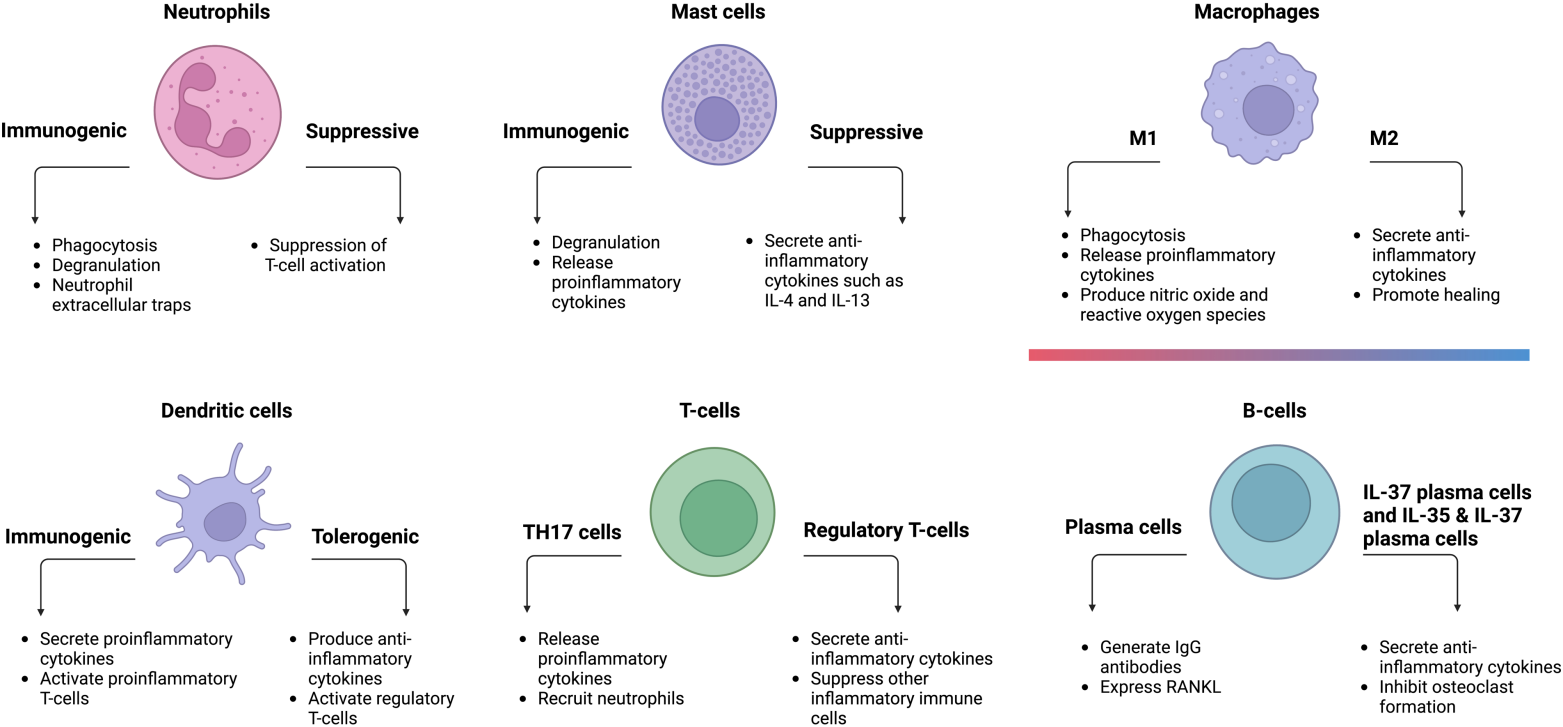
Different types of immune cells and their respective functions in periodontitis.

In summary, periodontitis is a multifactorial disease caused by interactions between bacteria and their by-products, immunomodulatory cytokines, and different immune cell populations (Figure 2). Furthermore, immune cells also communicate with one another via cytokines or cell–cell interaction (105). Elevated levels of neutrophils, plasma cells, and naïve B-cells were found in periodontitis-affected tissues. In terms of T-cells, periodontitis-affected tissues had a larger proportion of activated CD4 memory T-cells, whereas healthy tissues had a lower number of regulatory T-cells.

## 3. Biomaterials for modulating periodontal bone regeneration

Biomaterial scaffolds are three-dimensional constructs designed to support the proliferation and differentiation of cells for the regeneration of defective tissues (106). The optimal scaffold would be the extracellular matrix (ECM) of the native tissue. However, it is difficult to recreate the exact native ECM due to its intricacy and composition, as there are many types of ECM found in various human tissues. A biomaterial scaffold should serve several key functions similar to those of native ECM. First, the biomaterial scaffold should exhibit a highly interconnected porous structure to enable the transportation of nutrients and metabolites, hence facilitating the attachment or passage of cells and supporting their cellular activities. A porous structure also provides the necessary volume for vascularization and the development of new tissues. Second, the biomaterial scaffold should also be biocompatible and biodegradable. Biocompatibility refers to the absence of any serious adverse effects elicited by the biomaterial scaffolds. Biodegradability is another crucial factor since non-biodegradable biomaterials could face the challenge of foreign body reaction leading to the formation of a fibrous capsule, which may compromise the stability and functionality of implanted biomaterial scaffolds (107). Another advantage of degradable biomaterials is that they allow the newly formed tissues to eventually replace the degraded biomaterial scaffolds. Third, the scaffolds should possess similar mechanical properties to those of the native tissues in order to provide the proper mechanical cues to local cells. Previous studies have shown that scaffold surface morphology and stiffness can affect how cells differentiate (108, 109). Lastly, scaffolds may serve as a delivery vehicle for the sustained release of bioactive molecules, such as growth factors or therapeutic agents, to promote regeneration (110).

There are many ways to characterize biomaterials, one method is to classify them based on their structural and compositional complexity, mainly monophasic versus multiphasic scaffolds. Monophasic scaffolds have been extensively studied as potential delivery vehicles for biomedical applications. Although they are easy to fabricate and their interconnected porous structure promotes cell adhesion, they do not possess layers with distinct properties. In contrast, multiphasic scaffolds offer the advantage of having different layers with different characteristics such as material composition, architecture, and functionalization that allow them to target different tissues at once. For example, a triphasic collagen scaffold was developed by combining collagen self-assembly and diffusion gradients in mineralization (111). The resulting tri-layered scaffold resembled the architecture of the periodontium, with successive layers that mimicked the bone, periodontal ligament, and cementum, respectively. Each individual compartment can provide the necessary mechanical cues to help facilitate the regeneration of the corresponding tissue. Another study constructed a multiphasic scaffold composed of three different layers, each with its own specific microstructure and delivering a distinct bioactive cue that targets the regeneration of the cementum, periodontal ligament, and alveolar bone, respectively (112). Implantation of such a multiphasic scaffold into the dorsum of immunodeficient mice for 6 weeks resulted in the formation of a periodontium-like multi-tissue matrix. However, the main drawback of multiphasic scaffolds is that, in order to enable precise control of the microarchitecture of each layer, the scaffolds need to be prefabricated and then surgically implanted. This complicates the fabrication process, as different compartments need to be constructed differently. In addition, the geometry of the scaffold needs to be personalized based on the defect size.

Most often, for regenerative applications in the oral cavity, injectable biomaterials are preferred over their implantable counterparts. Injectable biomaterials are viscous formulations that are administered in a flowable state, either liquid or gel-like. This type of biomaterial includes but is not limited to hydrogels (113–115), cryogels (116, 117), supramolecular hydrogels (118), microspheres (119–121), cement (122–126), and so on. Their most distinctive advantage is that they can be readily administered through a needle, a simple and much less invasive procedure that minimizes collateral damage surrounding the injury site. Another advantage of injectable biomaterials arises from their viscoelastic property. Upon injection, the fluidic biomaterials can fill the irregularly shaped pockets associated with periodontal complications. However, to ensure injectability, the choice and design are often limited to biomaterials that are cross-linked physically via weak attractions, such as hydrogen bonding, electrostatic interactions, pie-pie interactions, van der Waals forces, and so on. Utilizing a dual-syringe system is one approach to circumvent this limitation; separation of the polymeric materials and cross-linkers maintains their respective stability; and, upon injection, the two components immediately mix and cross-link, resulting in a hydrogel with significantly stronger mechanical properties (127). Photopolymerizable biomaterials constitute an additional viable solution (128).

### 3.1. Biomaterials as delivery vehicles

The use of antibiotics is a common treatment option for chronic and aggressive periodontitis. Systemic administration of antibiotics has been proven to be effective at attenuating periodontitis by eradicating periodontal pathogens from deep periodontal pockets (129). However, systemic administration has several drawbacks. First, it is highly non-specific; administered antibiotics will circulate throughout the body, increasing the likelihood of adverse events occurring elsewhere. Second, overuse of antibiotics leads to bacterial resistance, which is becoming a global health crisis. Lastly, it is challenging to continuously maintain a high local concentration of antibiotics over time when they are administered systemically.

After systemic administration of metronidazole in rabbits with experimental periodontitis (130), the suspension was quickly detected in the blood after 6 h and the minimum inhibitory concentration was maintained for 24 h around the periodontal pocket. As opposed to systemic dosing, the sustained local release of antibiotics would be desirable. Biomaterials in the form of hydrogels, cements, micro-/nanoparticles, and microspheres are commonly utilized to deliver a variety of bioactive molecules and release them sustainably over an extended period of time (131) (Table 1). To enhance metronidazole distribution, a solution–gel-based inverse lyotropic liquid crystalline (LLC) system was developed. It gelled quickly, and its crystalline nanostructures improved adhesion to the periodontal pocket. Furthermore, the LLC sustainably released metronidazole, and its concentration was maintained above the minimum inhibition concentration for over 10 days without detectable concentration in the blood. Metronidazole was also delivered locally using an injectable adhesive chitosan and polyvinyl alcohol hydrogel in a comparable study (132). The hydrogel sustainably released the encapsulated metronidazole. In addition, it displayed excellent adhesion and prevented water seepage in a model tooth for 30 min. This feature can be exploited to seal the periodontal pocket and protect the periodontal tissues from further infection. In an *in vivo* rat periodontitis model, the mean probing depth of the periodontal pocket was significantly reduced in the hydrogel-treated group.

**Table 1:** Common biomaterial-based delivery system treatments for periodontitis.

Strategy	Biomaterial system	Animal model	Key findings	References
Local delivery to mitigate inflammation	Injectable adhesive hydrogel composed of chitosan decorated metronidazole microcapsules and poly vinyl alcohol hydrogel	Rat periodontitis model	Mean probing depth of periodontal pocket was significantly reduced in the hydrogel treated group	(132)
	Injectable hydrogel composed of amphipathic carboxymethyl-hexanoyl chitosan, β-glycerol phosphate and glycerol was constructed to deliver naringin	Murine periodontitis model	Hydrogel treatment considerably reduced alveolar bone loss and inflammatory infiltration relative to the untreated group	(133)
Local delivery to actively promote regeneration	Injectable macroporous calcium phosphate cement loaded with BMP-2/FGF-2	Rat periodontitis model	FGF-2 loaded injectable cement resulted in superior periodontal ligament and alveolar bone healing	(134)
	Injectable paste-like PLGA carrier was developed to encapsulate rhGDF-5	Canine periodontitis model	PLGA loaded with rhGDF-5 markedly regenerated cementum, periodontal ligament, and alveolar bone when compared to sham-surgery control	(135)
Local delivery to both subside inflammation and promote regeneration	Injectable self-healing hydrogel encapsulated with Rg1 and amelogenin	Rat periodontitis model	Hydrogel loaded with Rg1 and amelogenin significantly improved the quality of the regenerated alveolar bone	(136)
	Injectable thermosensitive hydrogel loaded with aspirin and erythropoietin	Rat periodontitis model	Hydrogel loaded with aspirin and erythropoietin reduced inflammation and promoted alveolar bone regrowth	(137)
Smart delivery system that is responsive to environmental cues	A smart on-demand delivery hydrogel was loaded with antimicrobial peptide and SDF-1	Rat periodontitis model	The smart hydrogel effectively controlled inflammation and facilitated bone regeneration	(138)

In addition to antibiotics, other therapeutic agents, such as statins and natural substances, have also been investigated for their efficacy to ameliorate local inflammation. Simvastatin is a statin commonly used to treat hyperlipidemia by decreasing elevated lipid levels. It also exhibits antioxidant properties, which may help reduce inflammation and support bone growth (139). However, its poor water solubility restricted its use in the oral cavity to treat periodontitis. To address this limitation, an injectable hydrogel was synthesized to load simvastatin and release it locally into the periodontal pocket (140). The loaded hydrogel resolved inflammation and preserved periodontal bone in a ligature-induced periodontitis rat model. Naringin is a flavonoid compound naturally found in citrus fruits, such as oranges, grapefruits, and bergamots, that has antioxidant and anti-inflammatory properties (141). Like simvastatin, naringin also has poor water solubility. One study synthesized an injectable thermosensitive hydrogel from amphipathic carboxymethyl-hexanoyl chitosan, β-glycerol phosphate, and glycerol to improve the oral delivery of naringin (133). The resultant hydrogel selectively released naringin between pH 5.5 and 6.5; it also significantly reduced alveolar bone loss and inflammatory infiltration relative to the untreated periodontitis group in a murine periodontitis model.

However, the main limitation of these studies is that they only targeted microbial dysbiosis-induced inflammation. As a result, this only reduced the extent of bone resorption but did not actively promote periodontium regrowth.

One approach to promoting tissue regeneration is by delivering regenerative molecules to directly facilitate site-specific regeneration. Injectable formulations of macroporous calcium phosphate cement loaded with bone morphogenetic protein-2 (BMP-2) or fibroblast growth factor-2 (FGF-2) showed periodontal regenerative potential. In a rat periodontitis model, the cement/FGF-2 group resulted in superior periodontal ligament and bone healing compared to the free cement and cement/BMP-2 groups (134). Injectable paste-like poly(lactic-co-glycolic acid) (PLGA) carriers have also been used to encapsulate recombinant human growth/differentiation factor-5 (rhGDF-5), a growth factor known to induce cartilage and bone formation (135). Following injection into the periodontal pockets in a canine periodontitis model, PLGA/rhGDF-5 was largely resorbed by the end of the second week. By week 4, there was a significant increase in the regeneration of cementum, periodontal ligament, and alveolar bone compared to the sham surgery control.

**Figure 3:**
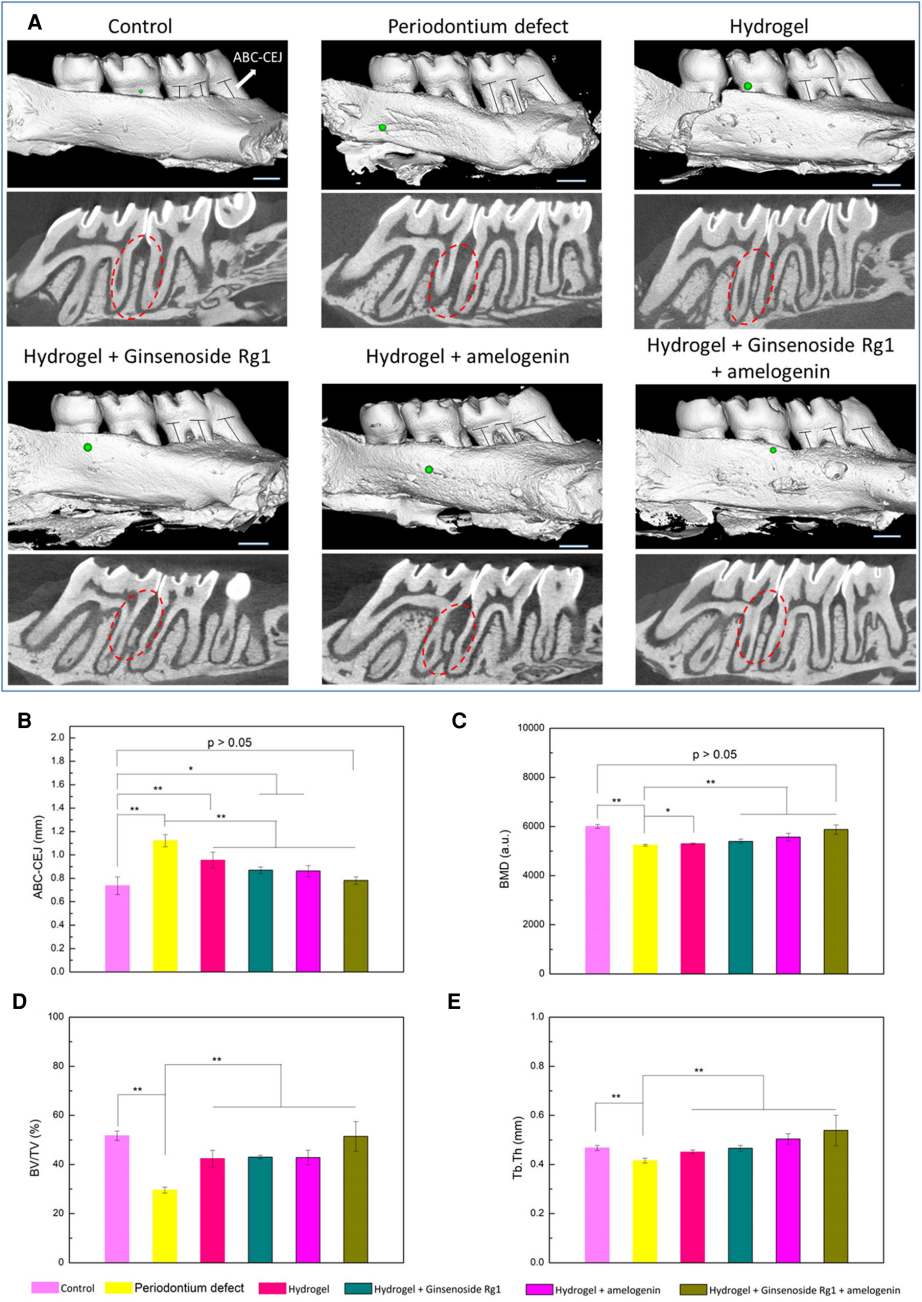
Evaluation of alveolar bone regeneration by micro-CT. (**A**) Three-dimensional and sectioned micro-CT images. Quantitative analysis of (**B**) the distance between ABC and CEJ, (**C**) BMD, (**D**) BV/TV, and (**E**) Tb.Th measured by micro-CT images. *p < 0.05, **p < 0.01. Scale bar is 1 mm. Reprinted with permission from (136). Copyright (2021) American Chemical Society.

Multiple bioactive molecules can be delivered simultaneously to achieve a synergistic effect to promote alveolar bone regeneration. A supramolecular hydrogel was utilized to improve the stability of stromal cell derived factor-1 (SDF-1) and BMP-2 (118). The delivery of the hydrogel loaded with these two bioactive molecules synergistically improved the bone regeneration outcome in a maxillary critical-sized periodontal bone defect rat model. Some studies have also focused on combination therapy to co-deliver both antibacterial agents and regenerative molecules to achieve a dual outcome. An injectable and self-healing hydrogel was constructed via a dynamically cross-linked network of dynamic Schiff base bonds and dynamic coordination bonds. The hydrogel was loaded with ginsenoside Rg1, a class of steroid glycosides that exhibits antioxidant properties, which also increases the proliferation activity and alkaline phosphatase activity of periodontal ligament cells (136), as well as amelogenin, a protein that promotes periodontal regeneration. The fully loaded hydrogel improved the quality of regenerated alveolar bone in terms of bone mineral density, bone volume/tissue volume, and trabecular thickness (Figure 3) (136). Similarly, a thermosensitive hydrogel loaded with aspirin (anti-inflammatory effect) and erythropoietin (promoting angiogenesis and bone regeneration) (137) suppressed inflammation and stimulated alveolar bone growth in a rat periodontitis model.

So far, it has been the case that most of the discussed injectable biomaterials release their encapsulated bioactive molecules by passive diffusion. While sustained release is achieved, the release kinetics are largely independent of environmental cues. Thus, it would be ideal to design a smart delivery system that only releases the encapsulated therapeutic agents in response to environmental cues to prolong the therapeutic window and reduce unnecessary release. In one study (138), a smart on-demand delivery system was engineered to only release the antimicrobial peptide in response to gingipain produced by *P. gingivalis*. The smart hydrogel was also loaded with SDF-1 to induce tissue regeneration. *In vitro* results demonstrated that the smart hydrogel had a sensitive response to gingipain stimulation and released the antimicrobial peptide in a controlled manner, which strongly inhibited the growth of *P. gingivalis*. In addition, the smart hydrogel effectively controlled inflammation, recruited stem cells, and facilitated bone regeneration in a rat periodontitis model.

These studies on biomaterials showed promising results. However, inconsistencies have been reported between different *in vitro* and *in vivo* studies. Because conventional regenerative strategies mainly promote osteoblastic cell differentiation, the treatment outcome depends on the elimination of pathogenic microorganisms, the stimulation of bone cells, and the host immune response.

### 3.2. Biomaterials for immunomodulation

Recently, it has been recognized that immune cells play a critical role in bone homeostasis, and there are complex interactions between the immune system and the skeletal system. This emerging field is known as osteoimmunology (142). Instead of focusing solely on osteogenic cells, this new approach has fostered advancement in developing biomaterials that target immune cells to indirectly promote bone regeneration, termed osteoimmunomodulatory biomaterials.

Compared to conventional biomaterial delivery systems, osteoimmunomodulatory biomaterials offer several advantages. First, immunomodulation of the local immune response could establish an anti-inflammatory microenvironment that is favorable to bone regeneration. Growth factors such as BMP-2 and platelet-derived growth factor-BB (PDGF-BB) have shown regenerative potential (143, 144). However, pro-inflammatory signals can hinder their regenerative activity, and an anti-inflammatory microenvironment could enhance their efficacy to induce superior regeneration (145). In addition, an immune system-initiated response is much more robust, as the modulated immune cells can secrete numerous immunomodulatory cytokines that can initiate a cascade of downstream cellular processes. It can be highly beneficial for immunocompromised patients when such biomaterials can provide a targeted approach. For instance, in diabetic patients with periodontitis, the pro-inflammatory microenvironment and the lack of regulatory immune cells following periodontal treatment can impair healing and bone regeneration. Therefore, apart from antimicrobial treatments, targeted site-specific immunomodulation can aid in diminishing the contribution of systemic inflammation associated with diabetes to the oral cavity.

Immunomodulatory molecules have been investigated to treat periodontitis. Pro-resolving lipid mediators are endogenous cell-signaling molecules released during inflammation to promote resolution of inflammation, aimed at restoring tissue integrity and function (146). They have shown to regulate many immune cells, such as neutrophils (147), macrophages (148), dendritic cells (149), T-cells (150), and memory b-cells (151). Lipoxin A4 is one of these mediators and its analog is associated with dampened neutrophils-mediated tissue degradation and bone loss in periodontitis (152). In one study, doxycycline and lipoxin A_4_ were loaded into a thermosensitive polyisocyanopeptide hydrogel (153). The hydrogel treatment significantly improved gingival clinical attachment than mechanical debridement alone in a canine periodontitis model. However, the immunological aspect was not investigated in this study.

More specifically, many published studies have mainly focused on the regenerative potential of M2 macrophages and regulatory T-cells (Table 2). They have been shown to create an anti-inflammatory environment that is favorable to bone regrowth.

**Table 2:** Common strategies of biomaterial-based immunotherapy for treating periodontitis.

Targeted immune cells	Biomaterial system	Animal model	Key findings	References
Macrophages	Controlled release of PLGA microparticles loaded with CCL2	Murine periodontitis model	CCL2-loaded microparticles decreased the M1:M2 ratio and established an anti-inflammatory microenvironment that prevented alveolar bone loss	(154)
	Liposomes loaded with resveratrol	Murine periodontitis model	Resveratrol-loaded liposomes reduced inflammation in the gingival tissues and ameliorated alveolar bone resorption, accompanied by an increased expression of M2 markers	(155)
Regulatory T-cells	PLGA microparticles loaded with CCL22	Murine periodontitis model/canine periodontitis model	CCL22 loaded microparticles decreased pro-inflammatory cytokines and reduced alveolar bone resorption in both the murine and canine periodontitis models	(156)
	PLLA nanofibrous spongy microspheres and mesoporous silica nanoparticles loaded with IL-2, TGFβ and miR-10a	Murine periodontitis model	Injectable formulation enriched and expanded regulatory T-cells locally and mediated against periodontal bone loss	(157)
	Microspheres were utilized to deliver IL-2, TGFβ, and rapamycin	Murine periodontitis model	Microspheres decreased alveolar bone loss by increasing the local population of regulatory T-cells	(158)
	Injectable hydrogel comprised of polyethylene glycol was utilized to deliver prolyl hydroxylase inhibitor	Murine periodontitis model	Hydrogel loaded with the prolyl hydroxylase inhibitor significantly elevated the local abundance of regulatory T-cells in the periodontal tissue and reduced alveolar bone resorption	(159)
Cytokine IL-17A secreted by IL-17 cells	Anti-IL-17A antibodies were incorporated into PLGA microparticles	Murine periodontitis model	The released antibodies from the PLGA microparticles inhibited alveolar bone loss and suppressed osteoclastic activity	(160)
Macrophages	Gold nanoparticle size (5–45 nm) can influence macrophage polarization	Rat periodontitis model	45 nm gold nanoparticles were effective at promoting M2 macrophages and they showed significant increase of bone formation and attachment	(161)
Uninvestigated	Injectable thermosensitive hydrogel composed of chitosan and β-glycerophosphate	Canine periodontitis model	Hydrogel promoted significant regeneration of periodontal ligament, formation of new bone and cementum without encapsulating any therapeutic agents. However, the hydrogel-host immune response was not studied	(162)

Various immunomodulatory cytokines have been encapsulated to induce M2 macrophages *in situ*. The C-C motif chemokine ligand 2 (CCL2), also known as MCP-1, was locally delivered by controlled-release microparticles to induce M2 macrophages (154). A decrease in the M1/M2 phenotype ratio and an anti-inflammatory microenvironment were established, which prevented alveolar bone loss in a murine periodontitis model. Similarly, liposomal systems loaded with resveratrol polarized macrophages from the M1 to the M2 phenotype, resulting in decreased levels of pro-inflammatory cytokines, such as IL-1β, IL-6, and TNF-α. In an *in vivo* murine periodontitis model, resveratrol-loaded liposomes reduced inflammation in the gingival tissues and ameliorated alveolar bone resorption, accompanied by an increased expression of M2 markers and a decreased expression of M1 markers (155).

Another commonly targeted immune cell population is the regulatory T-cells. PLGA microparticles were used to load and release a known chemoattractant for regulatory T-cells, CCL22 (156). In a murine periodontitis model, these PLGA microparticles containing CCL22 lowered pro-inflammatory cytokines and substantially reduced alveolar bone resorption. Furthermore, this formulation also reduced clinical measures of inflammation and lessened alveolar bone loss under severe inflammatory conditions in beagle dogs.

**Figure 4:**
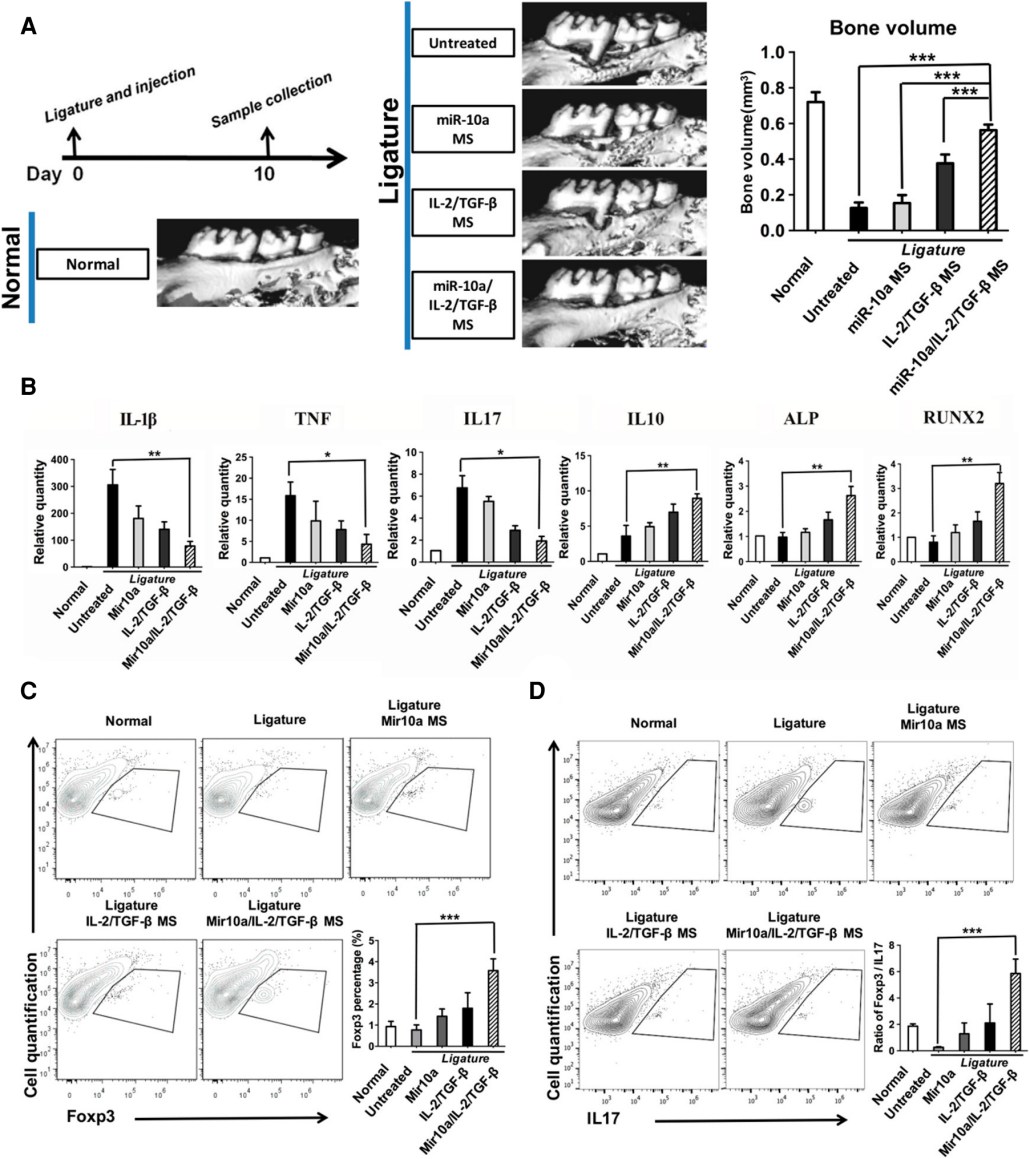
Multifunctionalized PLLA NF-SMS rescued bone resorption in a mouse periodontal disease model. (**A**) MicroCT results show bone loss between the first and the second molars in the periodontitis model and the bone volume changes in various treatment groups. (**B**) The gene expression of gingival tissues was quantified using real-time PCR, showing the effector T cell cytokines IL-1β, TNF, IL17, anti-inflammatory cytokine IL-10, and osteogenic markers ALP and RUNX2. (**C**) The flow cytometry analysis of the isolated gingival tissues shows the percentage of Foxp3^+^ cells (gated on CD4-expressing cells) in different treatment groups. (**D**) The IL17^+^ cells (gated on CD4-expressing cells) and the ratios of Treg/Th17 cells of the isolated gingival tissues are shown. Reprinted with permission from (157). Copyright 2018 American Chemical Society.

However, regulatory T-cells may become unstable and lose their anti-osteoclastogenic properties under prolonged periodontal inflammation (98). Therefore, the additional recruitment of existing regulatory T-cells to the oral cavity may not be an effective approach, as the systemic inflammation associated with periodontitis may have already compromised existing regulatory T-cells. Alternatively, regulatory T-cells can be induced from naïve T-cells *in situ*. In one study (157), a multibiologic delivery vehicle was fabricated by integrating nanofibrous spongy microspheres with functionalized mesoporous silica nanoparticles to release IL-2, TGFβ, and miR-10a to locally recruit T-cells and induce their differentiation into regulatory T-cells. In a murine periodontitis model, this injectable formulation successfully enriched and expanded regulatory T-cells locally, which mediated against periodontal bone loss (Figure 4). In a similar study, multiple bioactive molecules, including TGFβ, rapamycin, and IL2 were loaded into microspheres to induce regulatory T-cells locally (158). In a ligature-induced murine periodontitis model, the microsphere formulation decreased alveolar bone loss by increasing the local ratio of regulatory T-cells to effector T-cells. In a separate study (159), an injectable hydrogel was formulated to deliver the prolyl hydroxylase inhibitor, 1,4-dihydrophenonthrolin-4-one-3-carboxylic acid to promote regeneration of alveolar bone in a murine periodontitis model. Injection of the hydrogel induced an increase in bone regeneration, accompanied by an elevated expression of osteogenic genes, decreased expression of pro-inflammatory cytokine genes, and an increase in the number of FOXP3+ regulatory T-cells in the periodontal tissue.

Another approach is to target the major cytokines involved in periodontitis rather than the cells. The cytokine IL-17A plays an important role in periodontitis-induced bone resorption, and it has been identified as a possible treatment target. In one study (160), anti-IL-17A antibodies were incorporated into PLGA microparticles to neutralize IL-17A-mediated periodontal bone resorption. The delivery system sustainably delivered anti-IL-17A antibodies into the periodontium of mice with ligature-induced periodontitis, the released antibodies inhibited alveolar bone loss and suppressed osteoclastic activity (the anti-IL-17A formulation also decreased expression of IL-6, an IL17A target gene known to induce bone resorption in periodontal tissues).

However, the delivery of therapeutic agents faces other limitations, such as bioavailability and toxicity issues. Thus, it would be ideal to modulate immune cells using only the biomaterial scaffold's physicochemical properties. In one study (161), gold nanoparticles were synthesized with various diameters, including 5, 13, and 45 nm. *In vitro* cell culture results showed the 45 nm nanoparticles significantly increased the expression of Arg-1, IL-10, and TGFβ (markers of M2 macrophages). In a rat periodontal model, the 45 nm nanoparticles greatly enhanced bone formation and attachment while reducing tissue destruction. This could be attributed to the immunomodulatory effect of the nanoparticles, which promoted a macrophage phenotype switch from M1 to M2. In another study (162), autoclaved chitosan powder was dissolved and mixed with beta-glycerophosphate to formulate a thermosensitive hydrogel. In a canine periodontitis model, the hydrogel promoted a significant regeneration of periodontal ligament formation of new bone and cementum without encapsulating any therapeutic agents. However, the hydrogel-host immune response was not thoroughly explored. But based on the treatment outcomes, it is reasonable to postulate that the chitosan-based thermosensitive hydrogel exhibited an immunomodulatory effect that is favorable for regeneration.

## 4. Future considerations

Despite numerous publications on the development of novel biomaterial-based treatments for periodontitis, conventional treatments, such as scaling and root planing, and guided tissue regeneration remain the gold standard in the clinical setting. To better translate these scientific findings to the clinical setting, future research needs to take the following key factors into consideration.

### 4.1. Experimental periodontitis design and evaluation

The first hurdle that limits the translatability of periodontal research comes from the design of the experimental periodontitis model, as it is crucial to standardize the periodontitis model to allow better comparisons between different studies. The three most common experimental periodontitis models found in the literature are ligature-induced periodontitis, bacterial solution instillation-induced periodontitis, and ligature pre-soaked in bacterial solution-induced periodontitis. From the perspective of pathophysiology, it is necessary to include bacterial infection to study the interaction between dysbiotic microbiota and host immunity. Ligature-induced periodontitis, on the other hand, can rapidly establish the disease model by inducing bone loss (163). Thus, the most appropriate experimental periodontitis model would be the combination of bacterial instillation and ligature-induced periodontitis.

Equally important is the standardization of experimental parameters for evaluating treatment outcomes; currently, the primary method of assessing alveolar bone regrowth is via microcomputed tomography (micro-CT). It provides quantitative information about the extent of distribution of the bone mineralization at the macromolecular level of the bone structure, enabling the assessment of trabecular thickness and mineral density. In addition, a histological analysis, such as hematoxylin and eosin stain, is utilized to help visualize the local inflammatory response and the formation of other connective tissues, such as cementum and periodontal ligament. However, many published studies only focused on the healing and regeneration of the alveolar bone and neglected to investigate the regeneration of other connective tissues of the periodontium. Furthermore, immunohistochemistry can help characterize cell phenotypes based on surface markers and intracellular markers, and cells can also be isolated from the surrounding gingival tissues for gene expression analysis. In addition, other analyses, such as chemical analysis (byproducts released from the biomaterials) and mechanical testing (structural integrity of biomaterials post-implantation/injection), can also be performed to evaluate the biomaterial-based scaffolds, aside from treatment outcomes (164), to provide a complete assessment of the biomaterial scaffolds.

### 4.2. Animal model selection

The selection of an animal model is another important factor, with the challenge being that each animal model has its own set of advantages and limitations. Many animal models have been proposed to study periodontitis-induced bone loss (165). Non-human primates have similar tooth structures to those of humans and can naturally develop dental plaque, calculus, oral microbial infections, and periodontal disease on their own (166). However, they are expensive and have special ethical constraints, which have limited their use in periodontitis models. In contrast, although rats are inherently more resistant to periodontitis and their microbiota differ from that of humans, they are often used as an experimental periodontitis model because their periodontal anatomy in the molar region is comparable to that of humans. Furthermore, similar to humans, the bacterial instillation of *P. gingivalis* with ligature placement can successfully induce periodontitis in rats and result in alveolar bone loss (138, 161). More recently, immunodeficient mice that have been engrafted with human cells or tissues, commonly known as humanized mouse models, have been used to study periodontitis (167). In one study, subgingival tissues from periodontitis patients were engrafted into immunodeficient mice, and subsequently, the human periodontitis-associated bacteria efficiently colonized and further aggravated bone resorption (168). This new periodontitis model offers the opportunity to better understand the effect of human periodontal-associated microbiota in periodontitis and to develop innovative biomaterial-based treatments.

### 4.3. Treatment modality

Aside from standardizing the periodontitis design, evaluation, and model selection, it is also necessary to differentiate between the different treatment modalities, mainly prevention and treatment. Only studies that started the biomaterial treatment after the periodontitis model was successfully established should be considered as treatment. While studies that commenced the biomaterial treatment and ligature placement simultaneously before the periodontitis model was established should only be classified as prevention. For instance, in one study (140), a thermoresponsive hydrogel was administered concurrently with ligature placement in a rat periodontitis model. Despite the conclusion that the hydrogel formulation reduced inflammation and preserved periodontal bone, it should only be considered a successful preventive measure rather than an effective treatment since the periodontitis model was not fully developed when the intervention started.

## 5. Conclusion

In conclusion, the regeneration of periodontium is a complex process as it involves the regeneration of cementum, periodontal ligament, and alveolar bone. Currently, many studies have solely focused on the regeneration of alveolar bone alone by utilizing biomaterial-based therapy. Although some successes have been reported, it is important to point out that a newly formed alveolar bone could happen without the formation of a new cementum and periodontal ligament; instead, bone growth could be accompanied by the down growth of epithelial tissues into the periodontal defect (169, 170). Thus, regeneration of the alveolar bone alone does not necessarily constitute regeneration of the entire periodontium. The challenge lies in the complexity of the periodontium, as it is composed of both soft and hard tissues with differences in architecture, mechanical property, and composition. Therefore, fundamental studies should be conducted to investigate the lineages of progenitor cells that are involved in the regeneration of each individual tissue, along with the crosstalk of cells between these tissues. Currently, the majority of the injectable biomaterials being developed are monophasic; although multiple components may be incorporated, the final mixture is often homogeneous and lacks a gradient of properties. The future design of scaffolds should also take the multi-tissue interface into consideration, as cell differentiation is heavily regulated by local environmental cues. Hence, it may be helpful to design a scaffold with a gradient of properties that allows the regeneration of both soft and hard tissues.
